# Acute psychiatric admissions from an out-of-hours Casualty Clinic; how do referring doctors and admitting specialists agree?

**DOI:** 10.1186/1472-6963-6-41

**Published:** 2006-03-29

**Authors:** Trygve S Deraas, Vidje Hansen, Anton Giæver, Reidun Olstad

**Affiliations:** 1 Psychiatric Centre for Tromsø and Karlsøy, University Hospital of North Norway, Tromsø, Norway; 2 Institute for Community Medicine, University of Tromsø, Norway; 3 Institute of Clinical Medicine, University of Tromsø, Norway; 4 Tromsø Casualty Clinic, Tromsø, Norway; 5 Psychiatric Research Centre of North Norway, University Hospital of North Norway (UNN), Tromsø, Norway

## Abstract

**Background:**

Over the last decades there has been an increasing pressure on the acute psychiatric wards in Norway. The major contributor to psychiatric acute admissions at the University Hospital of North Norway in the city of Tromsø in 2001 was the GP-based Tromsø Casualty Clinic, only open out-of-hours. We explored all acute psychiatric referrals from Tromsø Casualty Clinic in 2001. The purpose of the study was to characterize the admissions and assess the agreement between the referring doctors and the hospital specialists according to the need for hospitalization, agreement on application of the law and the diagnostic evaluation to assess whether the admissions were appropriate.

**Methods:**

Retrospective, record based, descriptive study comprising 101 psychiatric acute referrals from the Tromsø Casualty Clinic to the psychiatric acute wards at the University Hospital of North Norway.

**Results:**

The specialists accepted all referrals except one, they mostly agreed upon the diagnoses suggested by the referring doctors and they mostly confirmed the application of the law.

Seventy-five percent of the admissions took place during weekends, public holidays or nighttimes. Diagnoses of psychoses or suicidal attempts accounted for 76 % of the total referrals. Substance abuse was noted for 43 %, and in 22 % of all admissions the patients had stopped taking their psychopharmacological medication. The police assisted the referring doctors in one third of all admissions, and was the legal representative in 52 out of 59 involuntary admissions. Thirty percent of the admissions were first- time admissions. Thirty-two percent of the hospital stays lasted for three days or less. Median length of stay was 6.5 days.

**Conclusion:**

The casualty clinic physicians and the hospital specialists mostly agreed in their evaluation of patients indicating that most of the admissions were appropriate. The police was more often involved in the involuntary admissions than intended in the law. The proportion of patients with substance abuse was significant. Alternative treatment strategies should be developed for non-psychotic patients in need of short-term stays.

## Background

In the psychiatric system of care there is a dynamic relationship between acute departments, the out-patient services and the primary care services. Changes in the out-patient services and primary care services influence the acute psychiatric admission rates [1,2]. The total number of beds in the Norwegian psychiatric health service has declined by 57 % from 1970 to 2002 [3]. In the same period, out-patient services and primary care services have increased greatly [3]. Nevertheless, the number of admissions per year and the number of people admitted have increased, and the length of hospital stay has decreased [4]. The majority of the psychiatric admissions are acute, but the reasons for the higher pressure on the acute wards are not well investigated. Many studies on acute psychiatry focus on patient characteristics, diagnoses, violence and treatment inside hospitals [5,6]. Less studies focus on the characteristics of the admissions, different admissions pathways and the system of care, including primary care [7-11]. These studies differ regarding to who refer most psychiatric acute admissions. Many countries accept self-referrals to hospitals, including acute psychiatric wards. In Norway, referrals to specialized care are obligatory and general practitioners (GPs) and the GP-based casualty clinics function as gatekeepers for all psychiatric referrals.

The Norwegian Mental Health Act [12] intends to reduce relatives' burden of being the legal representative demanding an involuntary admission. The public authorities (municipality chief physician, police, the social services and the prison administration) therefore carry the primary responsibility to apply for civil commitments. The law also intends to ensure that the legal representatives are well informed about the situation and that they demand the use of involuntary admissions independent of the referring doctor.

In a Norwegian study, casualty clinic-physicians referred 51 % of the patients involuntarily admitted for assessment and the patients' personal doctors (GPs) referred 22 % [9]. In a preliminary study, we found that Tromsø Casualty Clinic-physicians referred 43% of the acute admissions to one of the psychiatric acute units at University Hospital of North Norway in spring 2001. This acute unit had an ambulatory acute team.

In Northern Norway, with Tromsø as regional capital, the total number of beds-to-population in psychiatric institutions is significantly lower than for the rest of the country [3]. Hence the pressure on the acute beds might be even higher than in the rest of the country, and the need to know if the referrals are appropriate is urgent.

In 2001, Tromsø Casualty Clinic had 1375 consultations (9, 4 % of total consultations) with a diagnosis of the P-chapter (psychological) in the ICPC-1 clinical coding system. Approximately 9 % of those resulted in a referral to one of the three psychiatric acute wards at the University Hospital of North Norway, Tromsø, and almost 20 % of those with a diagnosis of psychosis were referred.

We studied all admissions to the three psychiatric wards at University Hospital of North Norway in 2001 from the GP-based Tromsø Casualty Clinic. We chose to study the casualty clinic referrals because a) the largest proportion of acute psychiatric referrals came from the casualty clinic out-of-hours, hence they induced a significant workload on the psychiatric acute departments and b) casualty clinic had less optimal working conditions compared to other sources of referrals. The purpose of the study was to assess the agreement between the referring doctors and the hospital specialists according to the need for hospitalization, agreement on application of the law and the diagnostic evaluation to assess whether the admissions were appropriate.

## Methods

The analyses included patients with permanent residence in the Tromsø municipality (60 086 inhabitants in 2001) referred out-of-hours from Tromsø Casualty Clinic to the acute psychiatric units at University Hospital of North Norway from January 1^st ^through December 31^st ^in 2001.

There were 101 referrals for admission (71 persons), of which 100 were accepted (70 persons). Based on a checklist, data were collected retrospectively from referral letters from the Tromsø Casualty Clinic and from the hospital electronic registration system.

### Variables registered were

#### 1) Patient characteristics

Age, sex, marital status, care for own children, employment status, name of personal doctor.

#### 2) Characteristics of the admissions

Time of admission, number of admission for the patient, use of the acute-team, legal basis for referrals and admission, assistance of the police.

#### 3) Patients' status

Diagnosis (according to different classification systems; ICPC-1 in referral letters and ICD-10 as discharge diagnosis) [13,14]. Information on substance abuse from referral letter and discharge diagnosis was grouped as to whether the information existed in none, one or two sources.

#### 4) Characteristics of the hospital stays

Length of stay and changes of the legal basis. Time of admissions was divided into admissions during a) weekends (Fridays after 10 pm onward to Mondays 8 am and Public Holidays), b) evenings (Mondays-Fridays 4 pm until 10 pm) when the ambulatory acute-team was on duty and c) night time (after 10 pm and before 08 am next morning, weekends excluded).

We collected data for 2001, the first year after the implementation of the new Norwegian Mental Health Act [12]. Data for each individual were aggregated and analyzed both on individual and admission level. Data were made anonymous and analyzed descriptively by SPSS 11.0 [15]. Differences between groups were tested with Chi-square test and Mann-Whitney U-test, statistical significance p < 0.05.

## Results

### Patient characteristics

Mean patient age was 32.0 years, range 18–65 years, median 29.2 years. Thirty-nine of the patients (n = 70) were men (55 %). Forty-three persons (n = 54) were unmarried, six were divorced, four were married and 51 lived alone (n = 58). Employment information (n = 39) showed that 32 were economically supported by various benefit systems and seven were employed. For fifty-eight patients we lack information on care for own children. The patients (n = 70) came from 34 out of 49 different personal doctors lists in Tromsø. Number between the brackets refers to the number out of 70 persons we obtained data on in the specific variable.

### Characteristics of the admissions

Table 1 shows characteristics of the admissions. We found no self-referrals from the actual area during the study period. In 22 of the 100 referrals, it was noted that the patients had stopped taking their medication before being referred.

The police were involved practically in the admitting process for 34 of the admissions, of which 24 were compulsory. Information concerning present substance abuse was noted for 21 of those. Information in referrals on substance abuse was distributed evenly regarding weekday and first time versus re-admissions. The acute-team was actively involved in three out of twenty-three admissions that occurred in their opening hours.

Thirty percent of the admissions were first time. Mean length of stay was shorter for first-time admissions (mean 9.2 days, SD 19.4) than for readmissions (mean 25.4 days, SD 58.1) (Z = -2,875, p = 0,004)

### Patients' status

**Figure 1:**
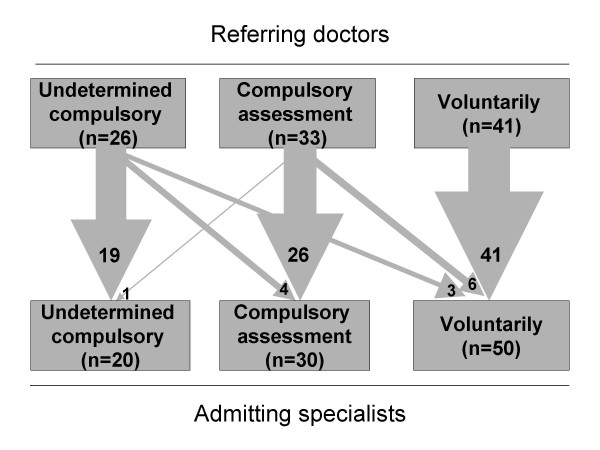
**Legal basis of admissions. **The legal basis of the acute psychiatric admissions from Tromsø Casualty Clinic in 2001 according to referring doctor and admitting specialist (psychiatrist or clinical psychologist) at University Hospital of Northern Norway. Numbers indicate number of patients.

The Norwegian Mental Health Act allows three different types of admissions: voluntarily, compulsory assessment (less than 10 days), and undetermined compulsory. The psychiatrist or clinical psychologist (hereafter named specialist) in charge either confirms or changes the legal basis within 24 hours. Figure 1 illustrates a high agreement between the referring doctors and the specialists when assessing the appropriate legal basis for admission. In only 14 out of 100 admissions, the admitting specialists changed the legal basis of the referrals.

Table 2 presents the diagnostic evaluation by the referring medical doctor (n = 96) and main diagnosis at discharge from the acute units. The majority of the referrals had a diagnosis of psychosis or suicidal attempt, according to ICPC-1 diagnosis list. The Tromsø Casualty Clinic- physicians used the diagnosis of psychosis more frequently than the specialists (predictive value of positive test 71 %), but the sensitivity of the Tromsø Casualty Clinic-physicians for diagnosing psychosis was 93 %. The diagnostic agreement between Tromsø Casualty Clinic-physicians and specialists were lower in non-psychosis cases, especially regarding personality disorders. Information on substance abuse was available in 43 admissions (Table 1). In only 19 of these, information was registered by both the referring doctor and the specialist.

### Characteristics of the hospital stays

Median length of the hospital stays was 6.5 days (mean 20.2, SD 48.5). In total, the 70 patients were hospitalized for 1993 days. Five patients accounted for 46 % of the days; all five were discharged with a diagnosis in the schizophrenia group. Information on substance abuse appeared in the referral letter for four of them.

Thirty-two hospital stays lasted three days or less, of which fourteen had a referral diagnosis of suicide attempt; eight had a referral diagnosis of psychosis (three confirmed at discharge) and the rest a non-psychosis or a symptom diagnosis. Sixteen of the short hospital stays were first time admissions; of these eight had a suicidal attempt diagnosis. Thirteen out of forty-three hospital stays with information on substance abuse had a maximum length of two days, and nine of those were first-time hospital stays.

## Discussion

We studied 101 referrals from the Tromsø Casualty Clinic to the psychiatric wards at the University Hospital of Northern Norway and found that the hospital specialists accepted all referrals except one and mostly agreed with the casualty clinicians on diagnosis and upon the application of the law. The police was more often involved in the involuntary admissions than intended in the law. The proportion of patients with substance abuse was significant.

Data were collected retrospectively from written sources. As a naturalistic study, information and assessments made by the doctors may be missing. Information on substance abuse, the role of the police and if the patients had stopped taking their psychopharmacological medication could be underestimated. Information on social background factors was insufficient. One the strength of our naturalistic study is the simplicity of the health care system we studied; there was only one casualty clinic and one psychiatric hospital, and no private sector.

### Agreement between referring doctors and hospital specialists

The processes leading to specialist care and how the GPs act as gatekeepers, vary between countries. A Dutch study documented that the number of self- referrals is much lower in areas with fully developed gatekeeper function [16]. Two studies on pathways to psychiatric care, focusing on acute psychiatric referrals, both show that GPs' referrals are accepted more often than self- referrals [8,17]. In Norway referrals to specialist care are obligatory. The personal list doctors (GP's) and the GP-based out-of-hours casualty clinics' doctors act as alternate gatekeepers for access to specialist care.

The doctors at the casualty clinic rarely were the patients' personal doctors, and the working conditions on the casualty clinic are not optimal for a psychiatric observation. Hence, some referrals from the casualty clinic could potentially be assessed as inappropriate at the in-hospital psychiatric examination. Interestingly, we found that all referrals but one from the GP-based Tromsø Casualty Clinic were admitted. We further expected that the referring doctors and the admitting specialists assessed the patients' condition differently, both regarding diagnosing and application of the law. Because the Norwegian Mental Health Act [12] allows a maximum time lag of 24 hours from admittance of the patient to verification of the legal basis, we anticipated that the possible time lag would cause a shift of the legal basis, because the patient would calm down after being hospitalized. However, we found that the legal basis was changed in only 15 % of the compulsory admissions and we consider the diagnostic agreement between the referring doctors and the specialist to be fairly good, especially for the psychotic patients.

A good gatekeeper should see to that all patients receive care at the right care level. We think the good agreement among referring doctors and specialists illustrates that the referrals were appropriate, and this may indicate that the casualty clinic doctors are good gatekeepers for acute psychiatric hospitalization. As almost all admissions were accepted we might further speculate that the casualty clinicians are too good gatekeepers and that too few patients are referred.

Nevertheless, to explore the appropriate threshold for referrals, and hence more thoroughly define the gatekeeper function, we need more information about patients not being referred. We also need information on other pathways to acute psychiatric admissions, especially referrals from personal doctors, to understand how the personal doctors handle acute psychiatric patients.

The proportion of referrals from the personal doctors was lower than those from the casualty clinic. The personal doctors can only be accessed one fourth of the 168 hours in a week. The personal doctors also have more available treatment and care strategies during daytime compared with the casualty clinicians, working evening and night shifts. Furthermore, factors leading to psychiatric emergencies such as substance abuse tend to escalate out-of-hours.

#### The role of the police

In our study the police was the legal representative in 52 out of 59 of the civil commitments. Seventy-five percent of the admissions took place when the municipality chief physician was inaccessible. As stated in the new Norwegian Mental Health Act [12] the police, as the social services and the prison administration, can request involuntarily hospitalization only if they are directly involved in the specific case. We find it reasonable that the police acted as legal representative when the police was directly involved in the referring and admitting process. In the rest of the involuntary admissions, the public health officer or her substitute would have been the natural choice of legal representative. Civil commitments procedures as they were practiced, raises questions about both legal safeguards and the quality of the professional assessment, since the police as legal representatives in our study had a passive role in most involuntary admissions. We expect that health professionals have a better basis than the police for decision-making in compulsory admission procedures. This would require a 24-hours on-call duty for public health officers, at least in cases where the police are not directly involved.

### Substance abuse

We found information on substance abuse in more than 40 percent of the admissions. The estimate is nevertheless probably too low. We expect that both the hospital's and the personal doctors' patient records would have given additional information.

Other studies indicate that substance abuse among psychiatric patients increases. A study from London demonstrated a two-fold increase of patients with drug problems from 1988 to1998, showing that 49 % of the patients had a history of substance misuse in 1998 [18]. A British study found that 44 % of Common Mental Health Teams patients reported past-year problem drug use and/or harmful alcohol use [19].

In a Norwegian study [20], substance abuse was relevant for the psychiatric condition for 54 % of the patients. Information of a substance abuse history might influence the choice of treatment and the interpretation of the current symptoms of the patients, at least for persons with earlier psychotic episodes [21]. Since comorbidity seems to be the norm rather than the exception, it is important to pay more attention to the patients' history of substance abuse. By performing a standardised interview most patients are found to give correct information on their history of substance abuse [20]. This is supported by the British study, where hair and urine analysis did not add any significant information to self-reported data.

### Short stays

In our study, the proportion of admissions with a short length of stay was rather high and more admissions were first-time admissions than shown in another study from Northern Norway [22]. As Hatfield et al, we found that most patients admitted were unmarried and lived alone without work [23]. This proportion was higher than reported in another Norwegian study [24]. Altogether this may indicate that more patients admitted from Tromsø Casualty Clinic than from other referring agents were a socially selected group with scarce social network.

### Implications of the study

Characterizing acute admissions to a psychiatric hospital might help to develop appropriate service delivery models. Our study focuses on the most important pathway to acute psychiatric care in Norway and concludes that most referrals from the casualty clinic were accepted indicating that the casualty clinicians are efficient gatekeepers for the acute psychiatric departments. On the other hand we do not know whether some patients in need of acute psychiatric hospitalization are denied access to the right care level. Our study implicates that civil commitments procedures as they were practiced, raises questions about both legal safeguards and the quality of the professional assessment, and call for better procedures. We underline the importance of increased focus on the substance abuse of the acute psychiatric patients. In our opinion, better alternatives to admissions in an overcrowded psychiatric acute department should be developed. Observation and detoxification beds, better psychiatric out-patient care, including extended opening hours in weekends for the ambulant acute-team, may improve quality of care for some of the patients.

## Conclusion

The casualty clinic physicians and the hospital specialists mostly agreed in the need for hospitalization, evaluation of the symptoms and the application of the law, indicating that most of the admissions were appropriate. The police was more often involved in the involuntary admissions than intended in the law. The proportion of patients with substance abuse was significant. Given the existing primary care services and the acute psychiatric service, we found that the GPs at the Tromsø Casualty Clinic referred patients with obvious need of acute psychiatric care, but alternative treatment strategies should be developed for non-psychotic patients in need of short-term stays.

## Competing interests

The authors declare that they have no competing interests.

## Authors' contributions

TSD initiated and planned the study. TSD and AG collected the data.

TSD, RO and VH carried out the data analyses and contributed to the writing of the manuscript. All authors read and approved the final manuscript.

## Pre-publication history

The pre-publication history for this paper can be accessed here:



## Figures and Tables

**Table 1:** Characteristics of 100 acute psychiatric admissions and hospital stays referred from Tromsø Casualty Clinic to the University Hospital of North Norway 2001.

**Characteristics**	N
**Number of admissions (N = 100)**	
First time admissions	29
Readmissions	66
No information	5
**Referring doctors main work place (N = 38)**	
Personal doctors*^1^* (GPs)	16
Internship doctors	6
Others	16
**Day of admission and time of the day (N = 100)**	
Weekends and Public Holidays	55
Weekdays	43
Evenings (Mondays to Fridays 4 pm to 10 pm)	24
Night time (10 pm to 08 am)	19
No information	2
**Length of hospital stays (N = 100)**	
≥ 3 days	32
4–7 days	21
8–14 days	17
15–21 days	12
21–28 days	5
≥ 29 days	13
**Legal representative (N = 59)**	
Police	52
Mother or father	4
Spouse	1
Municipals chief physician	1
No information	1
**Registered information on substance abuse (N = 100)**	
In referring letters or as discharge diagnosis	24
In both referring letters and as discharge diagnosis	19
No information	57

^1 ^Personal doctor; from 2001 a patient list system

**Table 2:** Diagnostic agreement. Referring doctors' diagnoses (ICPC- 1) compared to specialists' (psychiatrist or clinical psychologist) diagnoses (ICD- 10) at discharge

	**ICD-10 **Specialists' diagnoses at discharge (n = 96)						
	
**ICPC-1 **TCC-medical doctors' diagnoses at referral (n = 96)	Psychoses F20-29, F30.2, F31.2, F19.5 (n = 44)	Organic mental disorders F0-9 (n = 1)	Use of psychoactive substances F10-19 ^3 ^(n = 7)	Affective disorders without psychosis F30-39 ^4 ^(n = 15)	Neurotic, stress related, somatoform disorders F40-49 (n = 10)	Personality disorders F60-69 (n = 15)	Unspecified mental disorders F99 (n = 4)
Psychoses (n = 56)^1^	40	-	1	8	1	3	3
Symptom diagnoses (n = 10)^2^	3	1	2	2	1	-	1
Depressive condition P76 (n = 6)	-	-	-	2	2	2	-
Suicide attempt P77 (n = 18)	-	-	2	3	6	7	-
Personality disorders P80 (n = 4)	-	-	1	-	-	3	-
Mental disorder NEC/ NOS P99 (n = 2)	1	-	1	-	-	-	-

^1^P72 Schizophrenia all types, P98 Psychosis not otherwise specified (NOS), P73 Affective psychoses,

^2 ^P19 Drug misuse, P29 Psychological symptoms of complaints not elsewhere classified (NEC) /NOS,

^3 ^F10-19 minus F1X.5 -disorders with psychosis,

^4 ^F30-39 minus F 3X.2 -disorders withpsychotic symptoms

## References

[B1] Hansen V (1987). Psychiatric service within primary care. Mode of organization and influence on admission-rates to a mental hospital.. Acta Psychiatr Scand.

[B2] Catalano R, McConnell W, Forster P, McFarland B, Thornton D (2003). Psychiatric Emergency Services and the System of Care. Psychiatr Serv.

[B3] Hansen V, Oiesvold T (2004). Community psychiatry in the sub-arctic. Experiences with the shift from hospital-based to community-based psychiatric services in Northern Norway. Epidemiol Psichiatr Soc.

[B4] Norway S (2005). [Beds in psychiatric institutions and departments]. http://www.ssb.no/emner/03/02/speshelsepsyk/arkiv/tab-2002-09-18-01.html.

[B5] Munizza C, Furlan PM, d'Elia A, D'Onofrio MR, Leggero P, Punzo F, Vidini N, Villari V (1993). Emergency psychiatry: a review of the literature. Acta Psychiatr Scand Suppl.

[B6] Brabrand J, Friis S (1997). [Involuntary admissions in emergency psychiatric institutions. A comparison between the county of Hedmark and the Ulleval sector in Oslo]. Tidsskr Nor Laegeforen.

[B7] Steel RM, McKay IM (2000). Pathways to psychiatric admission: a study of 100 consecutive admissions to south Glasgow acute adult psychiatric wards. Health Bull (Edinb ).

[B8] Oiesvold T, Sandlund M, Hansson L, Christiansen L, Gostas G, Lindhardt A, Saarento O, Systema S, Zandren T (1998). Factors associated with referral to psychiatric care by general practitioners compared with self-referrals. Psychol Med.

[B9] Gjelstad K, Lovdahl H, Ruud T, Friis S (2003). [Compulsory admissions for observation in emergency psychiatric departments--discharge next day?]. Tidsskr Nor Laegeforen.

[B10] Tse SK, Wong TW, Lau CC, Yeung WS, Tang WN (1999). How good are accident and emergency doctors in the evaluation of psychiatric patients?. Eur J Emerg Med.

[B11] Bjorngaard JH, Heggestad T (2001). [Can case-mix explain differences in involuntary admissions?]. Tidsskr Nor Laegeforen.

[B12] Ministry of Health and Care Services (2001). Norwegian Mental Health Act.

[B13] Lamberts H, Wood M, Hofmans-Okkes I (1993). The International classification of primary care in the European community : with a multi-language layer.

[B14] WHO (1992). ICD-10, the ICD-10 Classification of Mental and Behavioural Disorders.

[B15] (2001). SPSS 110 manual.

[B16] van Uden CJT, Winkens RAG, Wesseling GJ, Crebolder HFJM, van Schayck CP (2003). Use of out of hours services: a comparison between two organisations. Emerg Med J.

[B17] Mackenzie DM, Mackie J (1993). Psychiatric emergency clinic attenders: what can we learn from them?. Health Bull (Edinb ).

[B18] Fitzpatrick NK, Thompson CJ, Hemingway H, Barnes T, Higgitt A, Molloy C, Hargreaves S (2003). Acute mental health admissions in inner London: changes in patient characteristics and clinical admission thresholds between 1988 and 1998. Psychiatr Bull.

[B19] Weaver T, Madden P, Charles V, Stimson G, Renton A, Tyrer P, Barnes T, Bench C, Middleton H, Wright N, Paterson S, Shanahan W, Seivewright N, Ford C (2003). Comorbidity of substance misuse and mental illness in community mental health and substance misuse services. Br J Psychiatry.

[B20] Helseth V, Lykke-Enger T, Aamo TO, Johnsen J (2005). [Drug screening among patients aged 17-40 admitted with psychosis]. Tidsskr Nor Laegeforen.

[B21] Curran C, Byrappa N, McBride A (2004). Stimulant psychosis: systematic review. Br J Psychiatry.

[B22] Høye A, Hansen V, Olstad R (2000). First-admission schizofrenic patients in northern Norway, 1980-95: Sex differences in diagnostic practice.. Nord J psychiatry.

[B23] Hatfield B, Spurrell M, Perry A (2000). Emergency referrals to an acute psychiatric service:Demographic, social and clinical characteristics and somparison with those receiving continuing services. Journal of Mental Health.

[B24] Hagen H (1987). [Patients in psychiatric institutions]. (In Norwegian)..

